# Tumeurs Stromales Gastro-Intestinales «GIST»: état des lieux et actualités à travers notre expérience portant sur 54 cas et une Revue de littérature

**DOI:** 10.11604/pamj.2017.27.165.7754

**Published:** 2017-06-30

**Authors:** Nezha Taoufiq, Asmaa Naim, Zineb Bouchbika, Nadia Benchekroune, Hassan Jouhadi, Souha Sahraoui, Abdelatif Benider

**Affiliations:** 1 Centre Mohamed VI de la Lutte contre le Cancer Casablanca, Maroc; 2 Université Mohamed VI des Sciences de la Santé UM6SS, Maroc

**Keywords:** Tumeurs stromales gastro-intestinales, GIST, épidémiologie, diagnostic, traitement, Gastrointestinal stromal tumors, GIST, epidemiology, diagnosis, treatment

## Abstract

Les Tumeurs Stromales Gastro-intestinales « GIST » sont une forme très rare de cancers du tube digestif appartenant à la famille des sarcomes. Le but de ce travail est d'établir le profil épidémiologique et évolutif ainsi que les difficultés diagnostique et thérapeutique de cette pathologie maligne prise en charge dans un pays en développement. Une Etude rétrospective étalée sur 8 ans de Janvier 2002 à Mars 2010, a été mené au Service de Radiothérapie et d'Oncologie du CHU de Casablanca (Maroc) ayant colligée 54 cas de tumeurs stromales gastro-intestinales. L'âge moyen de nos patients était de 55 ans. Le délai moyen d'évolution était de 11 mois (0-72 mois). La biopsie a permis de confirmer le diagnostic dans 14 cas et la chirurgie dans 40 cas. La principale forme histologique était fusiforme (92,6%). Les GIST dans notre série avaient une taille tumorale moyenne de 12,5 cm avec un C-Kit positif dans 52 cas. Le risque évolutif a pu être établi dans 47 cas dont 39 avaient un risque élevé. La chirurgie était le principal traitement des patients de notre sérieAprès un recul moyen de 31 mois, la moitié des patients évaluables de notre série (n=19) est en rémission complète maintenue, le tiers (n=13) est décédé alors que le quart (n=8) présente une récidive locale et /ou métastatique. Quoique les recommandations soient éditées pour la prise en charge de ces tumeurs, ces dernières soulèvent encore de nombreux problèmes aussi bien diagnostiques que thérapeutiques dans notre contexte.

## Abstract (en)

Tumors Gastrointestinal Stromal “GIST” are a very rare form of digestive tract cancers belonging to the family of sarcomas. The aim of this study is to establish the epidemiological profile, the diagnostic and therapeutic difficulties of this malignancy supported in a developing country. A retrospective study spread over 8 years from January 2002 to March 2010, was conducted at the Department of Radiotherapy and Oncology of Casablanca (Morocco) have collated 54 cases of Gastrointestinal Stromal tumors. The average age of our patients was 55 years. The average time of evolution was 11 months (0-72 months). The biopsy confirmed the diagnosis in 14 cases and surgery in 40 cases. The main histological form was fusiform (92.6%). GIST in our series had an average tumor size of 12.5 cm with a positive C-Kit in 52 cases. The risk of progression was established in 47 cases of which 39 were high risk. Surgery was the main treatment of patients in our study. After a mean fellow of 31 months, half of evaluable patients in our series (n = 19) is maintained complete remission, one third (n= 13) died while a quarter (n= 8) has a local recurrence and / or metastatic. Although the recommendations are published for the treatment of these tumors, these still present many problems both diagnostic and therapeutic in our context.

## Introduction

Les tumeurs stromales gastro-intestinales « GIST », sont des tumeurs mésenchymateuses se développant le plus souvent à partir des cellules de Cajal de la paroi du tube digestif, elles sont exceptionnellement extradigestives. Bien que les GIST soient les sarcomes les plus fréquents du tube digestif, elles ne constituent toutefois que 0,2% des tumeurs malignes digestives pour une incidence en Suède et en France estimée à environ 12-15/1 000 000 d'habitants par an [1, 2]. Elles se caractérisent par une surexpression de C-kit en immunohistochimie et par des mutations activatrices de récepteurs tyrosine kinase. Ces tumeurs ont un potentiel de malignité connu, et leur pronostic est corrélé à la localisation, la taille de la tumeur et l'index mitotique. La chirurgie est le traitement de référence pour les formes localisées. Les thérapeutiques ciblées, tel que l'Imatinib puis le Sunitinib, ont transformé la prise en charge et le pronostic des formes avancées et métastatiques.

## Méthodes

Il s'agit d'une étude rétrospective de 8 ans réalisée au sein d'un centre hospitalier universitaire d'Oncologie et de Radiothérapie (Ibn Rochd, Casablanca-Maroc). Une série consécutive de 54 cas de tumeurs stromales gastro-intestinales colligée au centre entre 1^er^ Janvier 2002 et 31 Mars 2010 a été étudiée. Les données ont été receuillies à partir des dossiers médicaux, des comptes rendus opératoire et anatomopathologiques. Ont été inclus dans l'étude épidémiologique l'ensemble des patients adressés au centre pour prise en charge d'une tumeur stromale gastro-intestinale confirmée histologiquement. Ont été exclus de l'analyse des résultats thérapeutiques et évolutifs les patients perdus de vue après la première consultation.Du fait des faibles effectifs , les statistiques réalisées sur cette série ont été uniquement descriptives.

## Résultats

### Déscription de la population

**Figure 1:**
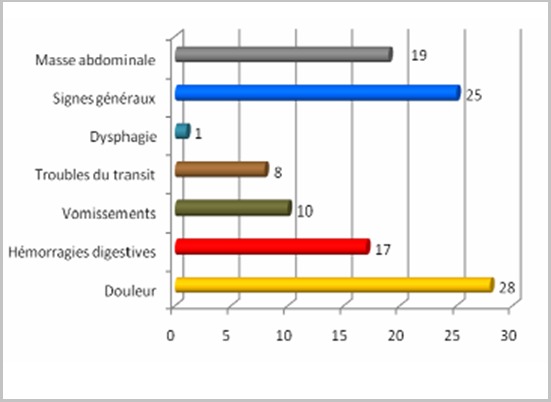
Graphique résumant la symptomatologie clinique des patients de notre série

L'âge moyen de nos patients était de 55 ans (23-80 ans) avec une fréquence plus élevée pour la tranche 40-60ans (61,1%). Une discrète prédominance masculine a été notée avec un sexe ratio de 1,16. La localisation la plus fréquente était gastrique dans 22 cas, succédée par l'intestin grêle dans 18 cas répartie comme suit: jéjunum (n=10), iléon (n=6) cas et duodénum (n=2). Notre série a recensé également: 6 cas de siège rectal, 4cas mésentérique, 3 cas coliques et un cas de GIST multifocale. Le délai moyen d'évolution était de 11 mois (0-72 mois). Seuls deux cas étaient de découverte fortuite, alors que les autres patients présentaient une symptomatologie clinique riche et intriquée dont le maitre symptôme était la douleur (Figure 1). La biopsie a permis de confirmer le diagnostic chez 14 patients: sous endoscopie dans 8 cas et par voie transcutanée écho ou scanno-guidée dans 6 cas. La chirurgie était le moyen de confirmation diagnostique chez 40 patients soit 74% des cas. L'étude histologique a montré une prédominance de la forme fusiforme (92,6%), tandis que la forme mixte et la forme épithéloîde ont été retrouvées respectivement chez 3 et un seul patient. La taille tumorale moyenne était de 12,5cm (2,5-27cm). L'index mitotique par champs (High Power Field: HPF) n'a été mentionné que chez 33 patients avec un HPF au-delà de > 5/50 dans 75,6% des cas. La C-kit effectuait chez tous les patients de notre série, était positive dans tous les cas excepté deux cas où la C-Kit était négative. La biologie moléculaire a été réalisée chez 2 patients et elle a montré dans les 2 cas une mutation de l'exon 11du gène C-kit. Le risque évolutif a pu être établi dans 47 cas (Tableau 1: récapitulatif de la répartition des GIST dans notre série). Au terme du bilan radiologique et histologique, le diagnostic de GIST localisée a été retenu dans 37 cas, localement avancée dans 8 cas et métastatique dans 9 cas.

**Tableau 1:** Récapitulatif de la répartition des GIST dans notre série

Estomac	22(40,7%)
Jéjunum	10(18,5%)
Iléon	6(40,7%)
Duodénum	2(11,1%)
Rectum	6(03,7%)
Mésentère	4(05,5%)
Colon	3(40,7%)
Multifocale	1(40,7%)
**Total**	**54**

### Prise en charge thérapeutique

**Figure 2:**
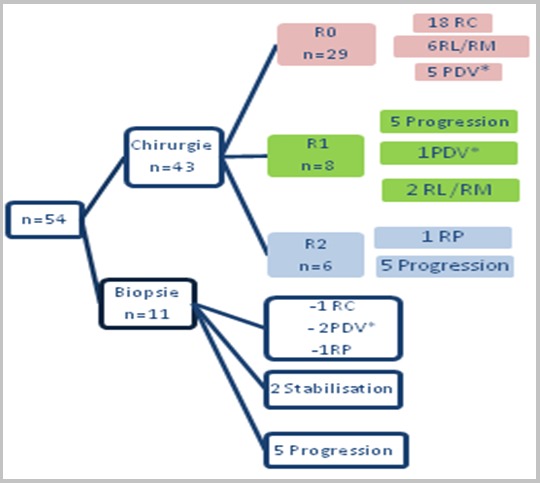
Graphique récapitulatif de l’évolution des patients de notre série

Un traitement néoadjuvant à base de chimiothérapie systémique, a été administré, avant la commercialisation de l'Imatinib dans notre pays, chez 4 patients. Les produits utilisés étaient l'Isofosfamide, l'Etoposide, le 5-fluoro uracile, l'Irinotecan, la doxorubicine et le cisplatine. Quant à l'Imatinib a été indiqué en néo adjuvant, à la dose de 400 mg/J chez 13 patients mais uniquement 10 patients l'ont reçu. La durée du traitement moyenne était de 10 mois (1-22 mois) et l'évolution a été marquée par un cas de rémission complète, deux cas de rémission partielle, une stabilisation chez deux patients et une progression chez cinq patients. La chirurgie était le principal traitement des patients de notre série, réalisait d'emblée chez 41 patients et après un traitement néo-adjuvant par Imatinib suite à une réponse partielle, dans deux autres cas. La chirurgie était de qualité R0 dans 29 cas, R1 dans 8 cas et R2 dans 6 cas. En adjuvant à la chirurgie, l'indication d'Imatinib a été posée en RCP, pour 32 patients hors seuls 20 d'entre eux l'ont reçu, pendant une durée moyenne de 12 mois (3-21 mois). L'évolution chez ces malades était marquée par le maintien de la rémission complète chez 10 patients, la rémission partielle dans un cas et la récidive locale et/ou métastatique chez 2 patients. Quant aux 7 autres patients, ils étaient en progression tumorale. L'administration de l'Imatinib, a induit des troubles digestifs (nausée + diarrhée) dans un cas, une réaction cutanéo-muqueuse isolée dans 2 cas et associée à des céphalées chez un patient et dans le cas restant une asthénie profonde avec oedemes et des troubles digestifs à type de nausées et vomissements. En fonction du degré et de la tolérance des effets secondaires, la prescription thérapeutique a consisté à additionner un traitement symptomatique (n=5), réduire les doses (1 cas) voir arrêter définitivement l'Imatinib (1cas). Le Sunitinib a été prescrit chez 4 patients suite à intolérance ou une progression sous Imatinib. Seuls 3 patients l'ont reçu, pendant une durée moyenne de 6 semaines. Après un recul moyen de 31 mois (2 à 106 mois), 15 patients de notre série étaient perdus de vue. Concernant nos patients évaluables (n=39), on note une rémission complète dans 48,7% des cas (n=19), une rémission partielle dans 5,1% des cas (n=2) et une stabilisation dans 2,6% des cas (n=1). De même le suivi a objectivé 13 cas décédés dont neuf suite à une progression tumorale (9/11) et deux secondaire à une récidive locale et/ou métastatique (Figure 2).

## Discussion

Les tumeurs stromales gastro-intestinales ou GIST sont des tumeurs rares: 1 à 3% des tumeurs malignes gastro-intestinales. Il s'agit de proliférations de cellules, le plus souvent fusiformes, parfois épithélioïdes, rarement pléiomorphes naissant dans la musculeuse du tractus digestif et exprimant le CD117 ou C-kit dans 90-95% des cas, le NSE dans 85-90% et le CD44 dans 60-80% [3]. La suggestion que la positivite au C-kit est requise pour le diagnostic de GIST est donc remise en question [4]. En effet, Il existe de rares exceptions, representant au maximum 5% des cas, où la proteine C-kit n'est pas detectable par immunohistochimie [5, 6]. Ces tumeurs peuvent être appelées GIST C-kit négatives ou plutôt« Tumeurs stromales à cellules fusiformes ou épithelioides compatibles avec une GIST». La tumorogénèse des GIST fait intervenir deux récepteurs: C-Kit et PDGFR. Les mutations de ces récepteurs sont hétérozygotes type gain de fonction [7, 8]. Le gène codant pour la proteine C-kit peut être siège de mutations dans 85% des cas et qui sont préférenciellement juxta membranaire (Exon11), peuvent également concernées la région extracellulaire de la protéine (Exon 9) plus rarement d'autres secteurs de la protéine, quant au gène PDGRF sa mutation est moins fréquente (10-15%). Dans notre série, l'incidence des GIST c-kit négatif est concordante avec la littérature (3,7%). Quant au profil mutationnel, une comparaison ne peut être faite vu que l'étude moléculaire ne s'est réalisée que chez deux patients. Les tumeurs stromales, sont rares avant 40 ans et exceptionnelles chez l'enfant avec un âge moyen de découverte entre 55 et 65 ans [9]. Il n'existe pas de prédominance nette de sexe, seules certaines études retrouvent une discrète prépondérance masculine avec une sex-ratio voisine de 1,5 [10–12]. Ces données concordent avec les résultats de notre série où l'âge moyen est de 55 ans et le sexe ratio Homme/Femme 1,16.

Les GIST peuvent se localiser dans tout le tractus digestif, la distribution topographique des GIST dans notre série ne rejoint pas parfaitement celle rapportée par la littérature. En effet, les GIST gastriques représentent la 1ère localisation mais à incidence plus faible dans notre série que celle de la littérature (40,7 % versus 60-70%), l'intestin grêle est la deuxième localisation aussi bien dans notre série que dans la littérature avec une fréquence quasi similaire (33,3% versus 20-30%). Notre série se distingue par la fréquence du siège colorectal trois fois plus importante que celle retrouvée dans littérature avec respectivement 16,7% et moins de 5%. La multifocalité des GIST reste exceptionnelle, un cas de notre série est une GIST multifocale. Les formes extra-digestives, sont rare représentent moins de 5% ; elles sont principalement mésentériques, des localisations intra hépatique [13] ou intra pancréatique ont été décrites [14, 15]. Dans notre série la seule localisation extradigestive est mésentérique avec une fréquence discrètement plus élevée (7,4%). Les particularités topographiques de notre série peuvent s'expliquer par un biais de recrutement. En effet, les patients adressés à notre structure présentent généralement des GIST localement avancées non résécables d'emblée. Il existe probablement d'autres cas, non référés où le traitement chirurgical exclusif était jugé suffisant. Les tumeurs stromales sont longtemps asymptomatiques, rendant leur découverte fortuite fréquente. Hors dans notre série, seuls 2 cas étaient de découverte fortuite. Ceci pourrait être justifié par le délai d'évolution de la symptomatologie avant la première consultation qui est en moyenne 11,2 mois (0-72 mois). La découverte à un stade métastatique a été similaire dans notre série à celle rapportée par la littérature avec respectivement 16,6% et 15-25% des cas [16]. Dans notre série, la douleur a constitué le maitre symptôme (51,8%) succédée des hémorragies digestives dans 31,4% des cas (n=17). Cependant, la revue de la littérature retrouve que les hémorragies digestives sont le plus souvent révélatrices des GIST (48% des cas) quant à la douleur sa fréquence n'excédent pas 36% des cas [17]. Les autres signes fonctionnels dépendent étroitement du siège de la tumeur.

Toute GIST est considérée comme potentiellement maligne [18] et devrait donc théoriquement être réséquée. Le curage lymphatique dans les GIST n'est pas réalisé de manière systématique [19, 20] car, comme pour les autres sarcomes, les GIST sont peu lymphophiles: le taux d'envahissement ganglionnaire est habituellement inférieur à 10 % et le risque de récidive ganglionnaire inférieur à 5 %. Il n'existe pas, contrairement aux autres sarcomes, de consensus sur la nécessité ou non d'un diagnostic préopératoire par ponction-biopsie (par voie écho endoscopique, percutanée ou opératoire) en cas de tumeur résécable. Le traitement chirurgical d'une tumeur stromale gastro-intestinale doit être macroscopiquement complet, sans effraction tumorale et avec des marges saines tout en privilégiant une exérèse fonctionnelle. En effet, les berges d'exérèse doivent être indemnes d'infiltration tumorale [19–21], mais il n'existe pas de consensus sur la distance de sécurité nécessaire entre le bord de la tumeur et la tranche de section chirurgicale. Cependant, une marge de 1à 2 cm est généralement considéré comme suffisante. Lorsque la lésion est résécable, un traitement néo-adjuvant par Imatinib n'est pas indiqué [19], par contre, l'Imatinib peut être indiqué après concertation pluridisciplinaire quand on estime qu'il peut modifier le geste opératoire en simplifiant la chirurgie ou en permettant une résection moins mutilante (préservation sphinctérienne pour le rectum par exemple). Dans notre serie, la chirurgie a été réalisé chez quarante trois patients, de qualité (R0) chez 29 patients soit 44,5% quasi similaire à la série Dematteo (40%) [22]. A noter que, deux cas dans ce groupe (R0) étaient jugés initialement non résécables, et qui ont répondu favorablement ( réponse partielle de 25 et 60%) au traitement néo adjuvant à base d'Imatinib reçu pendant une durée respectivement de 8 et 5 mois. Après un recul moyen de 31 mois (2-106 mois), l'évolution chez le groupe de patients (n=37) avec résection complète (R0+R1), était marquée par le maintien de la rémission complète clinique et radiologique chez 18 patients soit 48,6% concordant avec les données de la littérature à un délai médian de 2 ans (40-60%) [23]. Pour pallier à la fréquence des récidives, un traitement adjuvant s'impose. Hors, l'éfficacité de la chimiothérapie systémique dans les tumeurs stromales est faible, avec des taux de réponse de 0-10%. Cette chimiorésistance pourrait s'expliquer d'une part, par la forte expression des proteines de résistance aux anticancéreux (GlycoproteineP et la proteine de résistance multi drogue-1), d'autre part via le signal anti apoptotique émis suite à l'activation du C-Kit [24]. Par conséquent, l'action inhibitrice sélectif de la protéine c-kit que possède l'Imatinib et les résultats positives de l'étude *ACOSOG Z9001* justifie son utilisation en adjuvant dans les GIST c-kit positif à pontentiel récurentiel élevé. Afin de déterminer ce risque de récidive, le National Institute of Health (NIH) a proposé en 2002 une classification pronostique se basant sur deux criteres histologiques: la taille de la tumeur dans son plus grand diamètre et l'index mitotique pour 50 champs à fort grossissement [5]. En 2006, Miettinen a démontré en s'appuyant sur une large série de l'Armed Forced Institute of Pathology (AFIP) que le siège de la tumeur, pour une même taille et un même index mitotique, est également un facteur pronostic. Ainsi les GIST grêliques auraient un pontentiel de récidive plus important que celles gastriques [25, 26] .

Dans notre série, le risque de récidive, selon les 2 classifications AFIP et NHI a été élevé chez 39 patients et modéré dans 6 cas. Alors que dans 2 cas, le risque était modéré selon la classification AFIP et élevé selon la classification NHI. Pour les sept cas restants, on n'a pas pu conclure un risque précis de recidive car on ne dispose pas de l'index mitotique mais étant donnée leur siège et leur taille dépassant 10 cm , ils seraient de risque élevé ou modéré selon les 2 classifications. Dans notre série, l'Imatinib a été indiqué en adjuvant à la chirurgie chez 32 patients, seuls 20 d'entres eux l'ont reçu, défaut de moyens financiers. L'évolution de ce groupe de patients (n=20), était marquée par le maintien de la rémission complète dans la moitié des cas (n=10), une réponse partielle estimée à 65% s'est observée dans un cas. Alors que l'évolution était défavorable dans les autres cas (n=9), avec une progression tumorale chez sept patient et une récidive locale et métastatique dans deux cas après un délai de 8 et 17 mois. Cette non réponse à l'Imatinib pourrait être en rapport avec une résistance, sans pouvoir écarter l´eventualité d'un problème d'observance. En effet, la résistance à l'Imatinib, peut se traduire par une progression soit au début du traitement (<6mois) dite « résistance primaire » de l'ordre de 10 a 15 %, soit plutard (>1 an) dite « résistance secondaire » dans environ 15% [23]. Cette évolution clinique est surtout liée au profil mutationnel de la tumeur. Ainsi, la recherche mutationnelle, en plus de son rôle diagnostique, a un intérêt pronostique et prédictif de la réponse aux thérapies anti- C-kit. En effet, les taux de réponse sont de 83,5% pour l'exon11 versus 47,8% pour l'exon 9 [27] et la survie sans progression à 24 mois est à 69% pour l'exon 11 versus 14% pour l'exon 9 [28]. Par conséquent, la dose recommandée d'imatinib si mutation sur l'exon 9 est d'emblée 800 mg/j [29]. Certes, la tolérance de l'Imatinib est dose dépendante, néanmoins la majorité des effets secondaires sont souvent d'intensité modérée et régressent généralement au cours du traitement. Les trois effets secondaires les plus fréquement rapportés dans la littérature sont les oedèmes, l'asthénie et les troubles digestifs [30, 31]. De même chez nos patients, la tolérance était globalement bonne avec 16,7% des effets secondaires, n'ayant nécessité l'arrêt définitif de l'Imatinib que dans un cas. Le traitement standard, des patients developpants une résistance primaire ou secondaires ainsi que ceux présentants une intolérance à l'Imatinib qui s'avère rebelle au traitement symptomatique, est le Sunitinib. Ce dernier, est un inhibiteur aussi bien des recepteurs PDGFB que ceux de la VEGF. Il possède donc, une puissante activité antiangiogenique outre son activité antitumorale directe.Son efficacite a été demontrée par une étude de phase III multicentrique publiée en 2006, avec un gain en survie sans progression dans le bras Sunitinib (6,4 mois vs 1,5 mois ; p< 0,0001) [32, 33]. D'autres thérapeutiques ciblées tels que le Nilotinib, le Sorafénib et le Mastinib sont en cours d'essai [34, 35]. Dans notre serie, seulement 3 patients ont été traités par sunitinib pour une durée moyenne de 6 semaines, empêchant l'évaluation de l'efficacité du traitement.

## Conclusion

Les avancées spectaculaires de la biologie moléculaire, vont conduire certainement dans un futur proche à un traitement personnalisé et définitif des tumeurs stomales gastro-intestinales. Entre temps,dans notre contexte, le niveau socio-économique constitue un véritable obstacle pour le clinicien limitant la qualité de prise en charge thérapeutique. Toutefois, des efforts considérables sont en cours afin d'améliorer l'accès aux thérapies ciblées pour les patients à revenus modestes.

### Etat des connaissances actuelles sur le sujet

- Tumeur mésenchymateuse rare du tube digestif potentiellement curable (Rémission complète 60-70%);
- Rare avant 40 ans, ckit positif >95%;
- Localisation gastrique la plus fréquente.

### Contribution de notre étude à la connaissance

- Localisation colorectale 3 fois plus fréquente que dans la littérature (16.7% vs 5%);
- La découverte fortuite est souvent retrouvée dans la littérature hors elle est rare dans notre série;
- Fréquence de la douleur maitre symptome dans notre série alors que l'hemorragie digestive est la principale symptomatologie rapportée dans la littérature.

## Conflits d’intérêts

Les auteurs ne déclarent aucun conflit d'intérêt.
